# Identification of brazilein type B as a key marker for the presence of brazilwood in colored historical objects†

**DOI:** 10.1039/d5ay00798d

**Published:** 2025-08-01

**Authors:** Laura Hendriks, Rémi Martinent, Céline Spack, Gaëlle Bourgnon, Agnieszka Woś Jucker, Cyril Portmann

**Affiliations:** a School of Engineering and Architecture, Institute of Chemical Technology, HES-SO University of Applied Sciences and Arts Western Switzerland Pérolles 80 1700 Fribourg Switzerland laura.hendriks@hefr.ch; b Abegg-Stiftung Werner Abeggstrasse 67 3132 Riggisberg Switzerland

## Abstract

The identification of brazilwood in historical artefacts is challenging and strongly relates to the chosen extraction method. When strong acid hydrolysis is used, redwood's primary dyeing component, brazilein, is never found, but instead leads to the identification of two related marker compounds. Commonly referred to as type B and type C compounds, they are characterized by specific UV and mass spectra. While type C has recently been recognized as urolithin C, the molecular structure of type B has only been inferred and never elucidated. In this work, a combination of synthesis, UV spectroscopy, high-resolution MS and NMR spectroscopy was used to unambiguously determine the previously unreported structure of the brazilein type B derivative. These results support the long-standing hypothesis that an HCl-based extraction protocol leads to the formation of a dehydro-brazilein product. In addition, studies were conducted to evaluate the effect of the extraction conditions on the presence or absence of the marker compounds brazilein and brazilein type B. It was demonstrated that only brazilein type B is detected under strong acid conditions. Finally, this knowledge was used for the unequivocal identification of brazilwood markers in the study of four Italian red velvets using a milder HCl protocol, providing a historical perspective on the use of redwood dyes in Italy during the Renaissance.

## Abstract 2

The identification of brazilwood in historical artefacts is challenging and strongly relates to the chosen extraction method.

## Graphical abstract

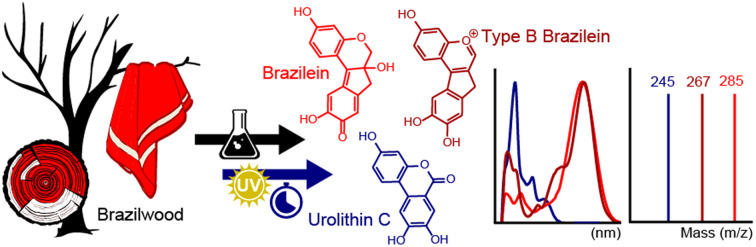

## Introduction

Throughout the course of history, the source of red colour has significantly extended and changed, from ground minerals such as earth pigments to organic dyes extracted from natural sources to a variety of synthetic colorants. Natural red dyes may be distinguished in two compound classes: anthraquinone derivatives and neoflavonoids. The former may be found in plant sources of the Rubiaceae family or from scale insects belonging to the Coccidae family.^1^ The second class, also known as soluble redwoods, is extracted from the red timber of various species of the genus *Caesalpinia* collectively termed brazilwood.^1^ Countless terminologies have been employed to denote the latter, generally associated with their geographical provenance as none are native to Europe.^2,3^ Sappanwood (*Caesalpinia sappan* L.), also known as Eastern Brazilwood, was imported from India and Southeast Asia since the early medieval ages.^4^ Pernambuco wood (*Caesalpinia echinata* L.) was introduced to the European market following the discovery of the New World.^5^ The principal colouring matter extracted from the red-colored timber is brazilin (1). This neoflavonoid is characterized by a poor colouring power and readily oxidizes on exposure to air to form brazilein (3), known as the main red-orange chromophore.^6^ A similar reaction is observed with logwood, where the main dyeing component haematoxylin (2) oxidizes to hematein (4).

Soluble redwoods have been an important source of red since the Middle Ages, whether for dyeing textiles or as a pigment in painted works of art.^7,8^ Despite a known poor lightfastness, their cheapness and ease of use made them an economically attractive source in comparison to other higher quality organic reds such as madder or cochineal.^9^ Nowadays, the original intended red hue, whether found in archeological textiles or sought in the brush stroke of artists such as Raphael, Rembrandt and Van Gogh, has long since faded.^10–15^ Exceptions are illuminated manuscripts, where the chromophore was protected from light exposure.^16–18^

Owing to the variety of substrates, a number of techniques have been employed for the identification of the redwood dyestuff, including both spectroscopic and chromatographic means.^8^ Over the years, for detailed dye analysis, Reversed-Phase High-Performance Liquid Chromatography coupled to Diode Array Detectors (RP-HPLC-DAD) has established itself as the method of choice for the routine analysis of natural organic dyes.^19^ Prior to HPLC analysis, the organic dye must be extracted from its matrix and solubilized. In the traditional approach proposed by Wouters and Verhecken, the samples are treated with a mixture of H_2_O : MeOH : 37% HCl (1 : 1 : 2, v/v/v) for 10 minutes at 100 °C.^20,21^ Under these harsh acidic conditions, the analysis of neoflavonoid dye molecules is not trivial, as they readily decompose.^22^
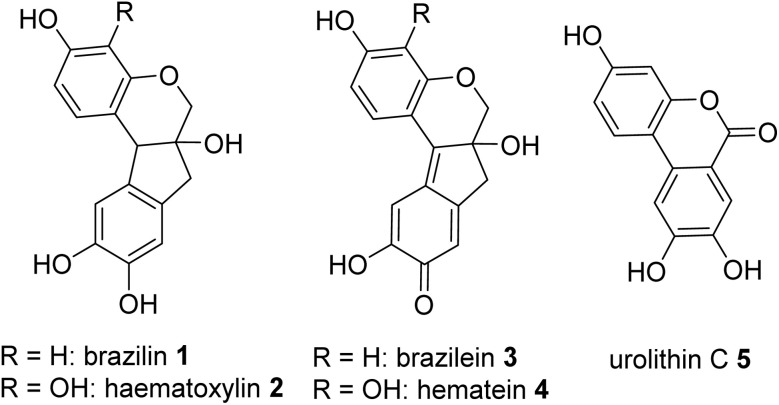


As such, brazilein (3) is rarely identified in historical objects due to its sensitivity to both acid and light, similar to hematein.^23^ The use of brazilwood, whether as a textile dye or as an organic lake pigment, is identified by the presence of an unknown brazilein derivative, also known as Bra', SRW or type B, together with another yellow compound, known as type C.^24^ The latter, a photodegradation product, was identified by Peggie *et al.* (2018) as the benzochromenone urolithin C (5).^25^ To date and to the best of the authors' knowledge, no study has reported the structure of the Bra', SRW or type B marker, although some hypotheses have been made. In 2005, Quye *et al.* reported hematein (4), brazilein's analog, and identified the logwood marker's dyestuff as its dehydrated counterpart.^22^ In their concluding statement, they suggested that a similar mechanistic step takes place when brazilein (3) is treated with hydrochloric acid. In 2009, in their work on Cretan icons and post-Byzantine textiles, Karapanagiotis' research group reiterated the hypothesis that the type B marker compound could correspond to the dehydro-brazilein product.^26^ Supported by ESI-MS data, they observed that in the absence of HCl, only brazilein (3) (*m*/*z* 283) is present, whereas the use of HCl-based protocols leads to the formation of the type B compound (*m*/*z* 265). Based on the Δ*m* = 18, they suggested that the type B compound, similar to hematein, is brazilein's dehydrated counterpart; however, they were cautious regarding the chemical structure. As a result, the characteristic UV spectrum with maxima at 237, 257, 322, 384 and 450 nm reported in Nowik's earlier work,^24^ together with the ESI-MS data corresponding to *m*/*z* 265 presented by the group of Karapanagiotis,^26^ has become the norm used by countless researchers as guidance in identifying brazilwood dye markers owing to the confusion surrounding the chemical structure of brazilein's type B marker.^13,15,27–34^

In this study, we report the structure determination of the brazilein type B derivative with a combination of synthesis, UV/VIS spectroscopy, high-resolution MS and NMR spectroscopy. The results support the hypothesis that brazilein, upon HCl treatment, is dehydrated similarly to hematein. With each research group having its preference regarding the hydrolysis extraction protocol, we furthermore explored how the sample preparation parameters (acid strength, temperature and time) influenced the dehydration reaction leading to the identification of brazilein or its dehydrated analogue. Finally, these results were put into context by analyzing historical silk velvets from the 14th to 16th centuries.

## Experimental section

### Instrumentation

NMR spectra were recorded on a Bruker 500 MHz Console Advance III with a BBO 500 MHz S2, 5 mm, with Z-gradient, PLUS SP probe, running on TopSpin 3.6.2. IR spectra were recorded on a FT-IR Bruker ALPHA in ATR mode. Acquisition parameters covered a spectral range from 4000 to 400 cm^−1^ with a resolution of 4 cm^−1^ at 32 scans. The melting point was determined using a Büchi Melting Point M-560 instrument. Mass spectrometry analyses were performed on an Exploris™ 240 FTMS instrument (Thermo Scientific, Bremen, Germany) operated in the positive mode coupled with a chip-based nano-ESI source (TriVersa Nanomate, Advion Biosciences, Ithaca, NY, U.S.A.) controlled by the Chipsoft 8.3.1 software (Advion Biosciences). Samples were solubilized in methanol and sprayed using an ionization voltage of +1.4 kV and a gas pressure of 0.30 psi. The temperature of the ion transfer capillary was 200 °C. FT-MS spectra were recorded in the 100–1000 *m*/*z* range with a resolution set to 120 000. MS/MS experiments were performed using an isolation window of 3 *m*/*z* in the 50–300 *m*/*z* range at a normalized collision energy of 80. The mass spectra were externally calibrated with the Pierce™ FlexMix™ calibration solution. Data analysis was carried out using XCalibur software (Thermo Scientific, Germany).

Chromatographic analyses were conducted on an Ultimate 3000 Dionex HPLC system (Thermo). Analyses were carried out by injecting 5 μL on an Adamas C18 HPLC column (100 mm × 4.6 mm i.d., 3.5 μm) from Sepachrom (Rho, Italy). The column was protected by a column guard, holding a C18 cartridge of 4 mm × 2 mm i.d. The compounds of interest were separated using a linear gradient. Solvent A – milliQ water + 0.5% formic acid and solvent B – acetonitrile. The gradient at a flow of 1 mL min^−1^ was 10% B to 100% B over 8 min. The column was then washed for 2 min at 100% B and stabilized for the next cycle over 6 min with 10% B. Monitoring wavelengths were 254, 280, 390 and 450 nm. Control of the HPLC system and data acquisition were performed using the Thermo Scientific™ Dionex™ Chromeleon™ 7 Chromatography Data System Version 7.2.10 Software.

### Materials

Standards of brazilin (98%) and brazilein (95%) were obtained from Cayman Chemical Company (Michigan, USA), while urolithin C (97%) was purchased from Sigma-Aldrich (St. Louis, USA). Milli-Q water was purified by an Aqua Max Ultra 370 series water purification system (Young In Chromass, Anyang, Korea). The following acids were used for sample preparation and chromatography: 37% hydrochloric acid from Carl Roth (Karlsruhe, Germany), oxalic acid dihydrate from Sigma-Aldrich (St. Louis, USA), orthophosphoric acid (85%, Lichropur) from Merck (Darmstadt, Germany), and formic acid (99%, Optima LC-MS) from Fischer Chemical (Reinach, Switzerland). Analytical grade methanol and acetonitrile from Carl Roth (Arlesheim, Switzerland) were used in all sample preparation and HPLC analysis. Brazilwood (*Caesalpinia sappan* L.), purchased from Maiwa Handprints Ltd. (Vancouver, Canada) and potassium aluminum sulphate dodecahydrate (99.5%) from Sigma-Aldrich (St. Louis, USA) were used for in-house dyeing of wool and silk cloths provided by the Abegg-Stiftung atelier.

#### Dyed textile mockups

The clothes were first scoured before mordanting for 30 min in an alum solution (6 g L^−1^, 500 mL) at 80 °C for 30 min. The brazilwood dyebath (3 g/500 mL water) was kept below the boiling point, and the textiles were immersed for 1 h at 80 °C. The dyed wool and silk clothes were rinsed with cold water, dried at room temperature and stored protected from light.

#### Historical samples

Four silk velvets dated between the 15th and 16th centuries from Italy were selected from the Abegg-Stiftung's collection. Both the main warp and weft thread of inv. no. 239 were sampled in the seam allowance of the upper left corner, as shown in Fig. 4. The orange-red weft thread of inv. no. 229 was sampled in the seam allowance of the upper left corner (Fig. S9c and d†). For object inv. no. 1690a, the orange-beige pile warp and weft thread were sampled in the upper left corner as documented in (Fig. S9a and b†). The fourth object, inv. no. 4329b, was also sampled for its orange-green pile chain at the seam allowance between the cuts from the reverse, whereas the red-brown weft thread was collected from an addition in the seam between the cuts from the back (Fig. S9e and f†).

### Synthesis

Brazilein (3) was prepared following a reported procedure.^6^ A suspension of *Caesalpinia sappan* L. brazilwood (30.2 g) in MeOH (200 mL) was stirred at room temperature for 2 days. The mixture was occasionally agitated with a glass rod. The dark red mixture obtained was filtered through a Buchner funnel. Solvent was removed *via* rotary evaporation until little MeOH remained and was left at rt for 3 days. The solution was then filtered, and small dark red crystals were washed with cold MeOH to yield brazilein (3) (100 mg, 0.3%) after drying. NMR signals were consistent with the reported literature.^35^

Brazilein type B (8) was synthesized following the reported procedure for the hematein analog.^22^ A suspension of brazilein (3) (76.5 mg, 269 μmol) in 37% aq. HCl/MeOH/H_2_O (2 : 1 : 1, v/v/v) (50 mL) was stirred at 100 °C for 10 min. Solvents were removed with a stream of nitrogen. Crude 8 (80.5 mg, quant.) was obtained as a dark red solid and was not further purified. NMR data in Table 1; mp: degradation over 200 °C; IR (cm^−1^): 3079 (br), 2917 (m), 2849 (m), 1629 (s), 1593 (s), 1503 (m), 1431 (m), 1368 (s), 1318 (s), 1229 (br), 854 (s); ESI-HRMS *m*/*z* 267.0652 [M + H]^+^ (calcd for C_16_H_11_O_4_^+^, 267.0652).

**Table 1:** ^1^H and ^13^C NMR spectroscopic data (500 MHz, CD_3_OD) for the brazilein type B (8) marker

Position	Type B brazilein (8) (CD_3_OD)^a^			
	*δ*_C_	*δ*_H_, (*J* in Hz)	HMBC^b^	NOESY
1	130.3, CH	8.75, d (9.2)	1a, 3, 4a 12	2, 11
1a	115.1, qC	—	—	—
2	122.1, CH	7.47, dd (2.4, 9.2)	1a, 4	1
3	168.8, qC	—	—	—
4	104.2, CH	7.35, d (2.4)	1a, 2, 3, 4a, 12	—
4a	160.7, qC	—	—	—
6	154.4, CH	8.96, s	4a, 12	7
6a	131.8, qC	—	—	—
7	34.1, CH_2_	4.13, s	8, 6a, 7a, 9, 12	6, 8
7a	159.1, qC	—	—	—
8	113.0, CH	7.25, s	7, 11a, 10, 7a	7
9	152.3, qC	—	—	—
10	149.9, qC	—	—	—
11	113.8, CH	8.00, s	7a, 9, 10, 12	1
11a	129.7, qC	—	—	—
12	163.9, qC	—	—	—

a ^1^H NMR data recorded at 500 MHz and ^13^C NMR at 126 MHz.

b HMBC correlations are given from the proton(s) stated to the indicated carbon. Chemical shifts (*δ*) are indicated in ppm and relative to TMS.

### Dye analysis protocol

Dyed reference materials were cut into clippings of 3–4 mg weight and treated with 500 μL hydrolysis solution upon heating in a Reacti-Therm™ heating module (Thermo Scientific, Massachusetts, USA). The small-scale hydrolysis reaction was quenched by immersion in an ice-water bath before evaporation to dryness under a nitrogen flow. The dried hydrolysates were reconstituted in a given volume of MeOH-0.1% H_3_PO_4_ and filtered through a 0.22 μm PTFE syringe filter (BGB Analytik AG, Böckten, Switzerland) prior to analysis. The traditional HCl based hydrolysis conditions (H_2_O : MeOH : 37% HCl (1 : 1 : 2, v/v/v) for 10 minutes at 100 °C) were modified with respect to hydrochloric acid concentration (10%, 5% and 2% HCl), temperature (60, 80 and 100 °C) and extraction time (10, 20, 30, 45, 60 min). Additionally, a milder approach using an oxalic acid (OA) hydrolysis solution,^36,37^ a mixture of methanol/acetone/water/0.21 M oxalic acid (30 : 30 : 40 : 1 v/v/v/v), was also tested (30, 60 min) at different temperatures (60, 80 and 100 °C). Samples were prepared in triplicate for each investigated parameter, and relative standard deviations were calculated.

## Results and discussion

### Identification and characterization of the brazilein type B (8) marker component

**Scheme 1:**
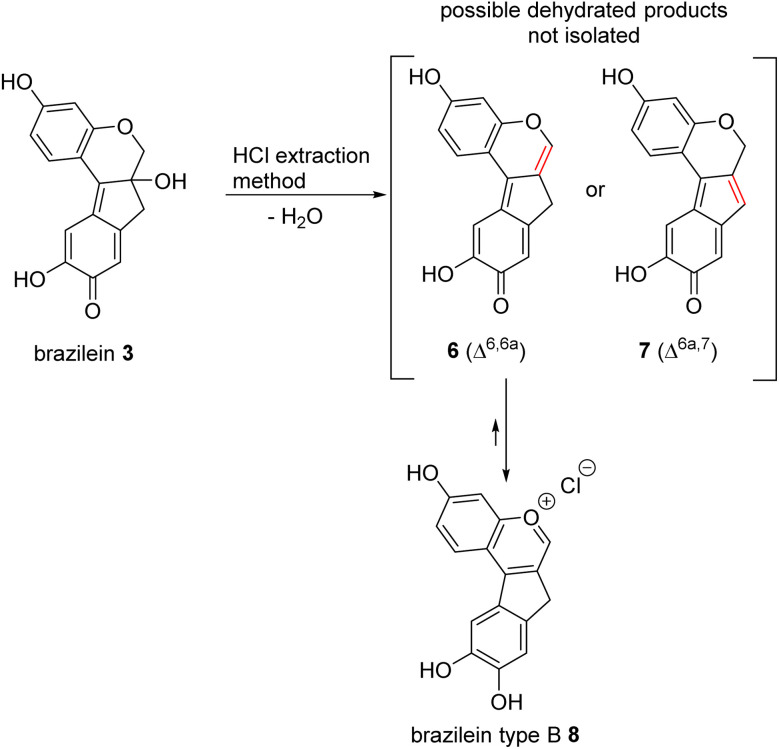
Structures of brazilein (3), the two possible dehydrated regioisomers Δ^6,6a^ (6) and Δ^6a,7^ (7) and the formation of the oxonium ion brazilein type B (8).

To confirm the long-standing hypothesis about the structure of brazilein type B (8), brazilein (3) was extracted from *Caesalpinia sappan* L. brazilwood following a standard protocol^6^ and was then dehydrated under acidic conditions following the same procedure as described by Quye and co-workers for Hematein dehydration (*i.e.* HCl conditions, Scheme 1).^22^ The formation of a dehydrated compound was observed, which was further identified as the previously reported brazilein type B marker based on the collected UV (Fig. S1†) and MS spectra.^24,26^

The high-resolution mass spectrum of the product displayed an exact mass of *m*/*z* 267.0652, which supports the molecular formula of C_16_H_11_O_4_^+^ for the ion (calcd 267.0652). The difference from the molecular formula of brazilein (3) corresponds to a characteristic loss of H_2_O followed by protonation.^35^

**Fig. 1:**
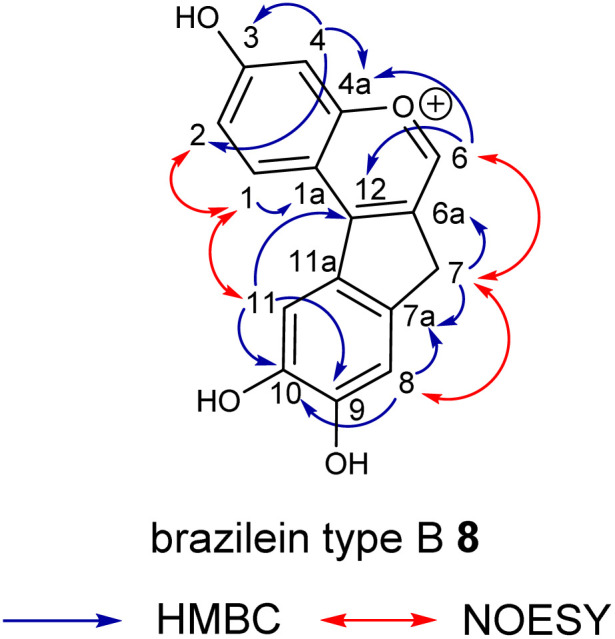
Key HMBC and NOESY correlations of brazilein type B (8).

The observed signal shifts in the ^1^H NMR spectra further confirmed the occurrence of a dehydration step (Table 1 and Fig. S2†). The disappearance of the H6 signals, originally two doublets at 4.03 and 4.56 ppm in brazilein (3), and the emergence of a singlet at 8.96 ppm in brazilein type B (8) are consistent with the elimination reaction. Moreover, the two diastereotopic protons in position 7 displayed a second-order signal at 2.84 ppm,^6^ which was replaced by a singlet at 4.13 ppm in 6 for two magnetically equivalent protons, further supporting the dehydration of the tertiary alcohol. The regioselectivity of the reaction was assessed by NOESY analysis (Fig. S4†). The structural isomer Δ^6,6a^ (6), similar to the elimination product of hematein (S9†),^22^ was supported by the through-space correlation between the 4.13 signal (H7) and the two signals at 8.96 and 7.25 ppm (H6 and H8 respectively, Table 1 and Fig. 1).

However, the deshielded chemical shift of the two protons in positions 6 and 1, 8.96 and 8.75 ppm, respectively, were inconsistent with structure 6. Additionally, the chemical shift of the tentative carbonyl in position 9 at 152.3 ppm was not consistent with the expected higher chemical shift for a carbonyl carbon. To explain these observations, it was postulated that brazilein type B, prepared under strong acidic conditions, formed the corresponding oxonium ion (8). Indeed, the p*K*_a_ of pyrone-like structures similar to 6 was calculated to be around 5.5.^38^ It is therefore plausible that under strong acidic conditions, the salt is obtained instead of the free base. The oxonium ion (8) is supported by the chemical shift of the two protons at 8.96 and 8.75 ppm (H6 and H1, respectively), which are similar to the chemical shift observed in isoflavylium salts.^39^

In addition, indirect observation supports the hypothesis that brazilein type B is in the form of a salt and not a free base. Firstly, brazilein type B is not soluble in many organic solvents such as chloroform or acetone and is only slightly soluble in methanol with partial degradation over time in solution, indicating that brazilein type B is a very polar compound. Secondly, if the free base 6 was obtained, it would be expected that the polarity of the compound would decrease compared to brazilein, and therefore yield a longer retention time of 6 on a reversed-phase HPLC analysis compared to brazilein. However, the retention time of brazilein type B was observed to be shorter than brazilein (see Fig. 4), indicating a more polar structure for brazilein type B. All these pieces of evidence support that brazilein type B is in the form of the oxonium salt (8). Based on the discussion above and the fact that the dehydration product of hematein (S9†) is obtained under similar conditions, and the similarity in the chemical shifts,^22^ it is possible that the dehydration product of hematein (S9†) (see Fig. S3†) is also in the form of an oxonium salt.

To the best of the authors' knowledge, this is the first unequivocal report of the structure of the brazilein type B (8) marker extracted from historical artworks upon strong hydrolysis.

### Impact of the hydrolysis extraction protocol on identified redwood markers

While acid hydrolysis in a methanolic solution remains the standard sample preparation protocol for historical textile dye analysis, the past decade has seen a proliferation of milder extraction methods to prevent hydrolysis-driven degradation of the investigated chromophores.^40^ The extraction protocols can hence be classified in two groups: harsh or mild acid conditions. As such, the effect of milder acids as well as time and temperature were investigated to elucidate the relationship between the two redwood markers, brazilein (3) and brazilein type B (8).

**Fig. 2:**
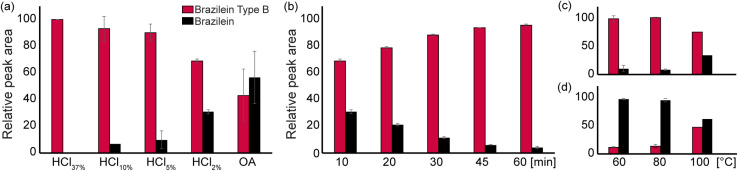
Relative integrated surface area monitored at 450 nm of brazilein (3) (black) and brazilein type B (8) (dark red) compounds extracted from silk samples dyed with brazilwood (*Caesalpinia sappan* L., after variation of (a) acid strength: HCl, 10 min at 100 °C and OA, 60 min at 80 °C; (b) time with 2% HCl at 100 °C; (c) temperature with 2% HCl for 10 min; and (d) temperature with OA for 60 min (*n* = 3).

The respective ratio of the two compounds (3 and 8) was monitored in dyed wool and silk mockups extracted under acidic conditions. As shown in Fig. 2, the acid strength and reaction time showed to have the most impact on the identified compounds in the analyzed dyed yarn hydrolysates. The bar plots represent integrated HPLC peak areas of both brazilein (3) and brazilein type B (8) compounds measured at 390 nm. When aq. 37% HCl is used to extract brazilein from the dyed yarn, only brazilein type B (8) is identified. In contrast, when “softer” extraction procedures are employed brazilein (3) is also observed. A decrease in the acid strength correlates with a decrease in the formation of 8. The use of oxalic acid is much less aggressive and allows the identification of 3 as the major compound and some 8. Although HCl-based protocols favor the dehydration reaction, shifting the equilibrium to the brazilein type B (8) product, the use of aq. 2% HCl allows the identification of both compounds. When conducted for 10 minutes at 100 °C, the ratio is close to 2 : 1 between compounds 8 and 3, which is displaced to the brazilein type B (8) side with longer reaction times. In contrast, temperature variations showed to have little influence in neither HCl or OA based extraction protocols. These results showed to be independent of the substrate, whether silk as displayed in Fig. 2 or wool in the ESI, Fig. S8.†

These observations correlate with what is reported in the literature and clarify why different laboratories report the identification of one or the other compound, depending on the chosen extraction method. This study establishes the link between brazilein type B (8) identification with HCl based extraction protocols. Milder conditions are essential to prevent/protect the neoflavonoid chromophore from degradation. These findings corroborates previous research where the identification of brazilein (3) as the major compound is reported when milder protocols are employed such as acetic acid,^23^ hydrofluoric acid,^41,42^ DMSO and formic acid,^43^ DMSO and oxalic acid,^15^ Na_2_EDTA/DMF^44^ or EDTA.^13,33^

While these recently developed milder alternative extraction protocols are successful in preventing undesired chromophore hydrolysis, their performance in terms of dye recovery are often not as satisfying as HCl based protocols.^19,44–47^ The limited sample availability in historical artefacts, the low concentration of the chromophore, and possible degradation due to aging are all parameters that require highly efficient extraction protocols to ensure the detection of the dyestuff's markers.^48^ The choice of the sample preparation protocol has thus important implications when considering the nature of the sample at hand. Harsh hydrolysis tends to produce higher yields, but often implies a complete degradation of the substrate.^19,44^ Moreover, the compounds resulting from fiber hydrolysis tend to deteriorate the quality of the chromatogram.^19^ A compromise may be found in the use of 2% HCl, which has been shown to produce similar results to the OA protocol in terms of fiber hydrolysis. The results collected for 2% HCl as a function of time show that the highest yield is achieved after 60 min. The importance of the sample preparation methodology and the deliberate choice of HCl-based over softer conditions, is reflected in the work of Shibayama *et al.* (2015),^30^ who preferred the use of 1 M HCl (∼3% HCl) to maximize the yield of extracted material and thus report the identification of brazilein type B (8) rather than brazilien (3).

Based on these observations, it was decided to use a milder extraction protocol for the analysis of the historical textile samples presented below. The conditions for the HCl based hydrolysis were H_2_O : MeOH : 2% HCl (1 : 1 : 2, v/v/v) for 10 minutes at 100 °C instead of the traditional 37% HCl.

**Fig. 3:**
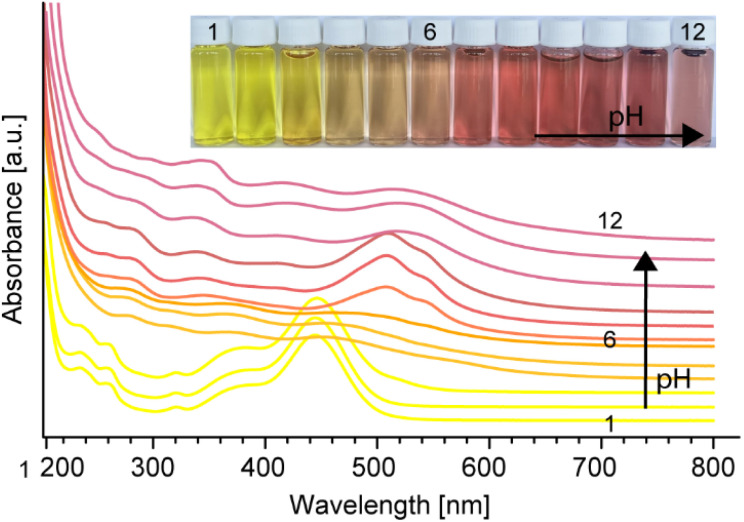
Color and respective UV-Vis spectrum variations of an aqueous solution of brazilein type B (8) under a stepwise pH increase from 1 to 12, adjusted with NaOH and HCl.

Not only is the hydrolysis step of interest, but the solvent used to re-solubilise the dry residue is also important. Similar to many red chromophores, brazilein type B (8) undergoes a color change as a function of pH, as shown in Fig. 3. Like brazilin (1) and brazilein (3), a yellow color is observed under acidic conditions, while a shift to red is observed following a pH increase into alkaline conditions.^49,50^ In an acidic environment the brazilein type B (8) species displays maximal absorption bands at 450 nm, with other absorbance bands at 237, 257, 322 and 384 nm as reported in Nowik's earlier work.^24^ With increasing pH, 8 exhibits a red color with a maximum absorption shifted to 510 nm and a minor absorption band at 340 nm.

### Brazilwood markers in Italian silk velvets

**Fig. 4:**
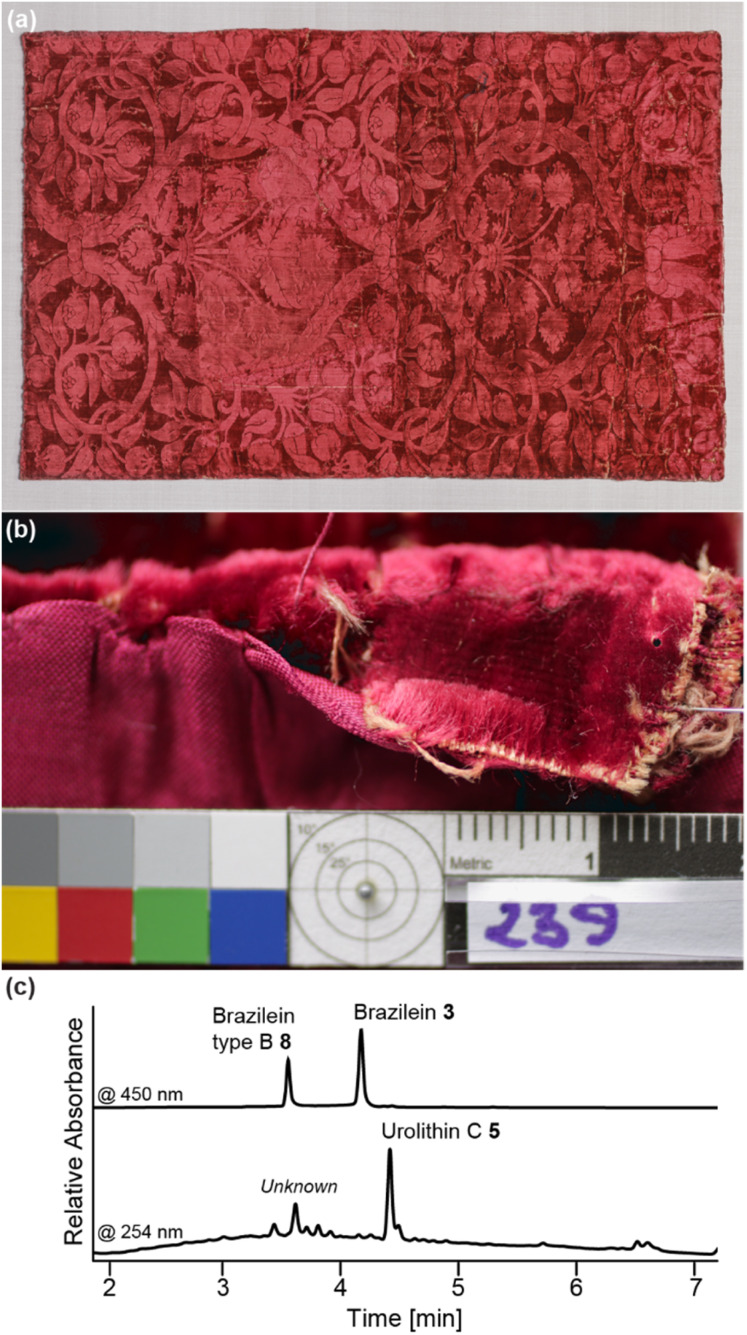
(a) Silk velvet fabric, Italy, early 16th century. Abegg-Stiftung, inv. no. 239. ©Abegg-Stiftung, CH-3132 Riggisberg, 2014 (photo: Christoph von Viràg). (b) Details to the sampling location of the main warp yarn (orange beige). ©Abegg-Stiftung, CH-3132 Riggisberg, 2023 (photo: textile conservation studio). (c) HPLC chromatogram of the extract of the warp yarn obtained with H_2_O : MeOH : 37% HCl (1 : 1 : 2, v/v/v) showing brazilein (3), brazilein type B (8) and urolithin C (5).

The aforementioned results were used to guide the interpretation of four silk velvets as an historical perspective regarding the use of redwood dyes in Italy during the Renaissance. The sampling of object inv. no. 239 is highlighted in Fig. 4, while all three other objects (inv. no. 229, 1690a and 4329b) are displayed in the ESI, Fig. S9.†

Italy has traditionally been recognized as the center of velvet production, where the patronage of various dukes in different cities highlights the economical and societal importance of the craft. The dyeing practices were highly regulated; however, they were only partially followed. Dye analyses have shown that despite being a lower quality red, brazilwood was ubiquitously used as a red dye source in silk velvet production in Italy.^51^ In this work we identified brazilwood markers in three out of four of the investigated Italian red velvets.

As shown in Fig. 4, the main color of the velvet stems from the pile warp, which according to previous examination indicated the use of polish cochineal with tannins. Here, we focused on both the main warp and weft of object no. 239. The sampled yarns showed a red beige hue, hinting to the still intact presence of brazilwood chromophores. All three markers were identified, namely brazilein (3), brazilein type B (8) and urolithin C (5). These findings highlight Italian dyers' practices, who as a function of the weave structure would select different quality red dyes sources.

The sole identification of urolithin C (5) in both the warp and weft thread of sample 1690a is consistent with the faded appearance of the velvet (Fig. S10a†). Despite an original intended red hue, urolithin C tends to show up in many archeological samples, as a result to light exposure.^13,15,52^

In addition to the brazilin's degraded marker urolithin C (5), the weft thread of object inv. no. 229 was also found to contain a higher quality red dye: madder (Fig. S10b†). The combined presence of alizarin and purpurin at 6.70 and 7.30 min, respectively, points to the use of *Rubia tinctorum* L.^32,53^ Prior analysis of the warp also indicated the use of redwood together with madder. Whether the identification of both dyes on each fiber is the result of cross contamination of dyes from adjacent threads, or whether both yarns were dyed with a mixture of the two dyes cannot be ruled out. Both hypotheses being valid as the cheaper redwood was often used for dyeing of the weft,^51^ while the combination of madder with redwood was commonly practiced as a substitute for the expensive kermes dye.^54^

The fourth item inv. no. 4329b is interesting as neither of the brazilwood markers were detected. Despite displaying a similar brownish hue as inv. no. 1690a, the main chromatographic peak at 3.45 min was identified as carminic acid, which is characteristic for cochineal species (Fig. S10c†). Carminic acid is considered of much higher quality than brazilwood as a red dye. It is found as main coloring matter in both Armenian and Mexican cochineal, which was introduced to the European market following the discovery of the New World.^4^ A distinction as to which of the dye sources was used may be performed based on the relative ratio of minor compounds such as flavokermesic and kermesic acid but these were not identified here.^21,55^ Based on the manufacturing date of the tapestry between 1460 and 1470, it was most likely Armenian cochineal that was used as dye source.

## Conclusion

In the analysis of archeological textiles, due to their poor light stability the presence of redwood neoflavonoids is predominantly indicated by the detection of the photodegradation product urolithin C (5). However, if protected from light some intact chromophores can be expected. The dye extraction protocol has hence a strong influence on the identified markers, as the neoflavonoids are not only light sensitive but also acid-sensitive, which complicates their analysis. The overall goal of this work was to understand the conundrum regarding the identified redwood markers associated with different extraction protocols. Our results indicate that the compound commonly known as the type B marker with *m*/*z* 265 and absorption bands at 237, 257, 322, 384 and 450 nm is indeed brazilein's dehydrated counterpart: brazilein type B (8). This compound is not inherently present in the sample but is instead artificially generated during the hydrolysis step required to release the dye from the matrix. Mild extraction protocols lead to the identification of brazilein (3) as the major compound, as milder conditions are essential to prevent/protect the neoflavonoid chromophore from degradation. In contrast, harsh extraction protocols, such as HCl-based, favor the dehydration reaction, shifting the equilibrium to brazilein type B (8) and its identification. Based on these observations and to maximize the yield of the extracted material, the hydrolysis conditions chosen for the analysis of the silk velvet samples were H_2_O : MeOH : 2% HCl (1 : 1 : 2, v/v/v) for 10 minutes at 100 °C instead of the traditional 37% HCl. Our findings provide valuable insights into the conversion of the target compound under different extraction protocols, highlighting the critical impact of preparation methods on analytical outcomes.

## Author contributions

L. H. and C. P. conceived the original idea. L. H. and R. M. coordinated the research. L. H. and C. S. developed the analytical sample preparation protocol. G. B. performed the dye analysis experiment. C. S. performed subsequent data analysis. R. M. carried out the synthesis and together with C. P. performed the identification and characterization of brazilein type B. L. H., C. S. and C. P. performed additional data characterisation and interpretation. A. J. provided resources under the form of study materials and art historical knowledge. L. H. coordinated the writing of the manuscript, with contributions from all authors. All authors read and approved the final version of the manuscript.

## Conflicts of interest

There are no conflicts to declare.

## Supplementary Material

AY-017-D5AY00798D-s001

## Data Availability

The data supporting this article have been included as part of the ESI.†
