# Supplementary material for: Identification of brazilein type B as a key marker for the presence of brazilwood in colored historical objects
Source: Anal Methods. 2025 Aug 1;17(36):7140–7. doi: 10.1039/d5ay00798d (PMC12315518; doi:10.1039/d5ay00798d)
Supplement: AY-017-D5AY00798D-s001 [file AY-017-D5AY00798D-s001.pdf]

Supplementary Information (SI) for Analytical Methods.
This journal is © The Royal Society of Chemistry 2025

Supporting information for the manuscript:

"Identification of Brazilein type B as a key marker for the presence of brazilwood in colored historical objects"

Laura Hendriks*‡[1], Rémi Martinent‡[1], Céline Spack[1], Gaëlle Bourgnon[1], Agnieszka Woś Jucker[2], Cyril Portmann[1]

[1]School of Engineering and Architecture, Institute of Chemical Technology, HES-SO University of Applied Sciences and Arts Western Switzerland, Pérolles 80, CH-1700 Fribourg, Switzerland

[2]Abegg-Stiftung, Werner Abeggstrasse 67, 3132 Riggisberg, Switzerland

This PDF file includes the following supporting information:

Figure S1 contains the collected DAD spectrum of brazilein type B (**8**)
Figure S2 compares the 1H-NMR spectrum of brazilein (**3**) against its dehydrated counterpart, brazilein type B (**8**)
Figure S3 structure of the dehydration product of hematein (**S9**)
Figure S4 displays the 2D NMR NOESY spectrum of brazilein type B (**8**)
Figure S5 represents the ^{13}C NMR spectrum of brazilein type B (**8**)
Figure S6. 2D HSQC NMR spectrum of brazilein type B (**8**)
Figure S7. 2D HMBC NMR spectrum of brazilein type B (**8**)
Figure S8 displays the relative ratio of brazilein (**3**) and brazilein type B (**8**) extracted from redwood dyed wool samples
Figure S9 displays the sampling of the 15-16th century Italian silk velvets from the Abegg-Stiftung's collection
Figure S10 summarizes the chromatographic analysis conducted on the sampled silk yarns

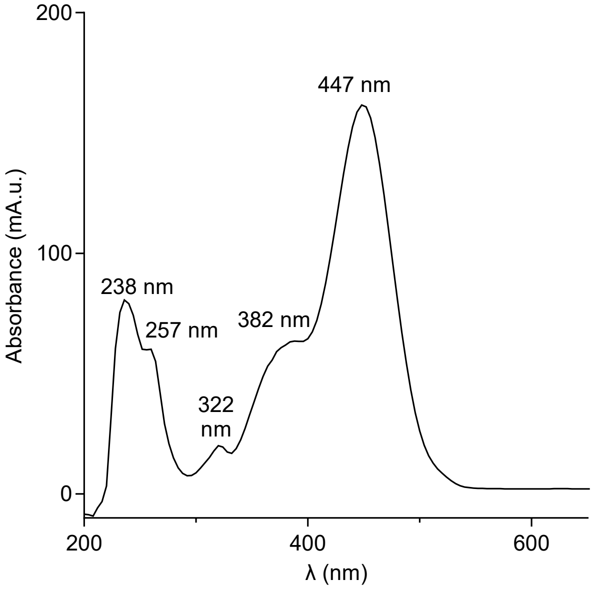

Figure S1. UV spectrum of brazilein type B (**8**).

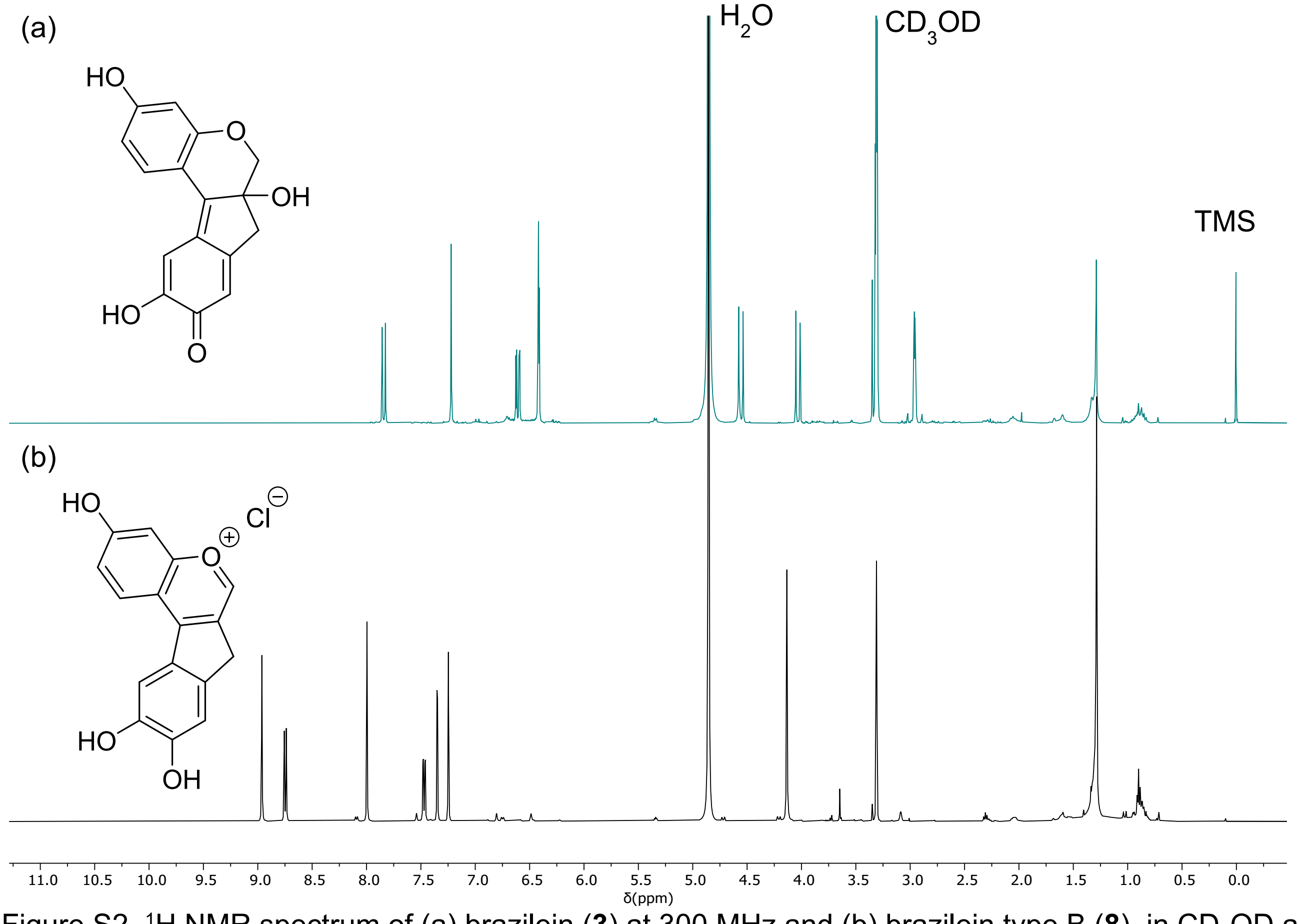

Figure S2. ^{1}H NMR spectrum of (a) brazilein (**3**) at 300 MHz and (b) brazilein type B (**8**) in CD_3OD at 500 MHz.

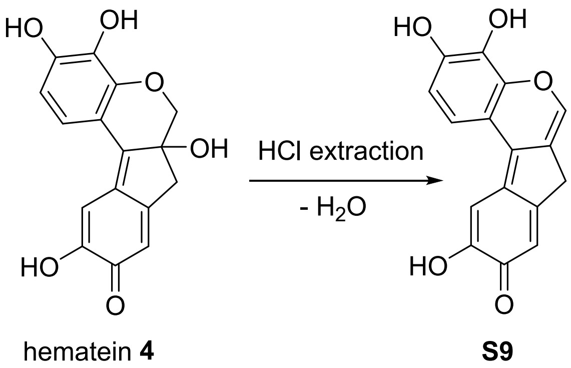

Figure S3. Dehydration reaction of hematein (**3**) to dehydration product (**S9**) as reported by Quye et. al.

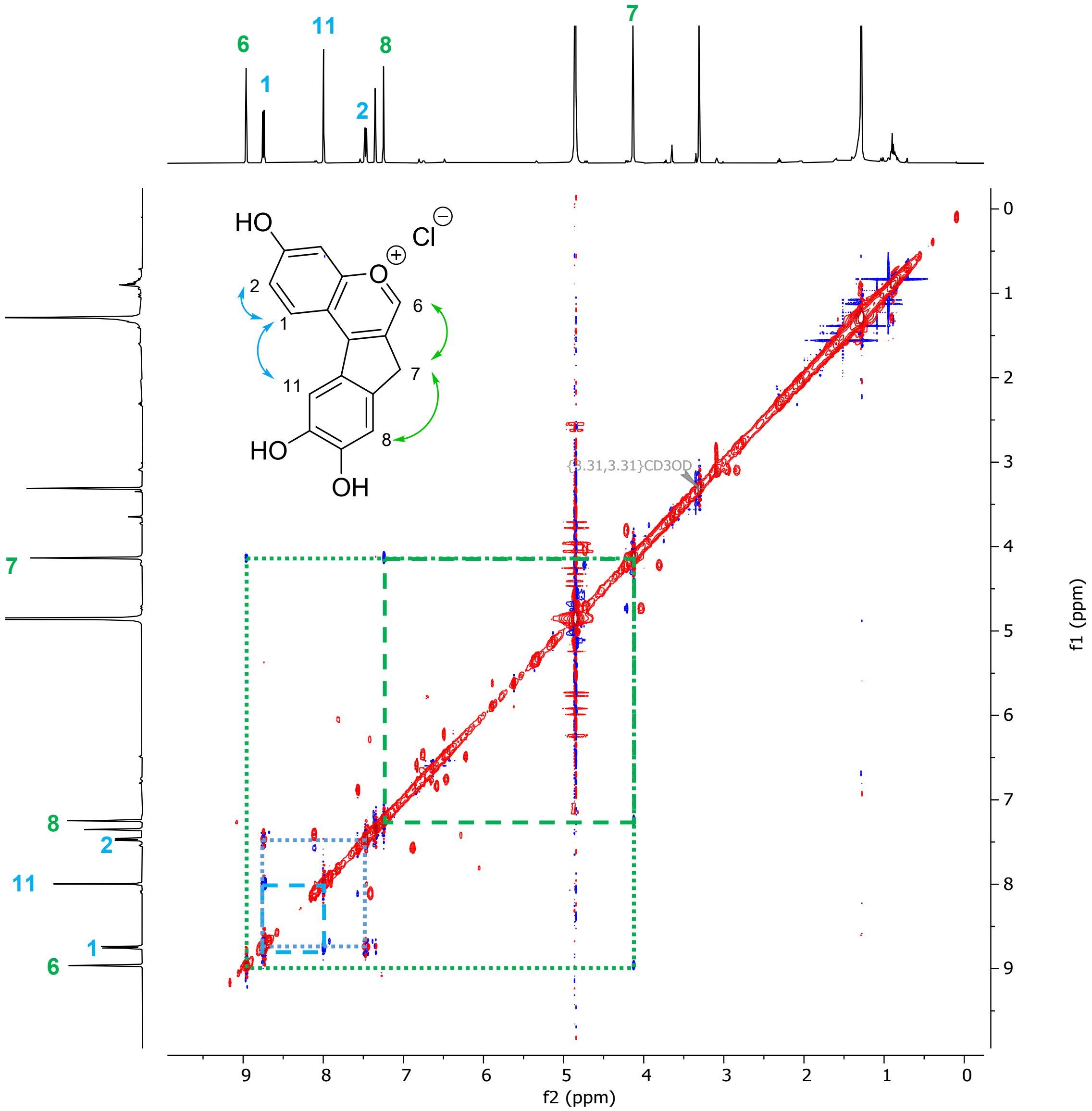

Figure S4. 2D NMR NOESY spectrum of brazilein type B (**8**) in CD_3OD (MHz). Main correlations are highlighted in green and blue.

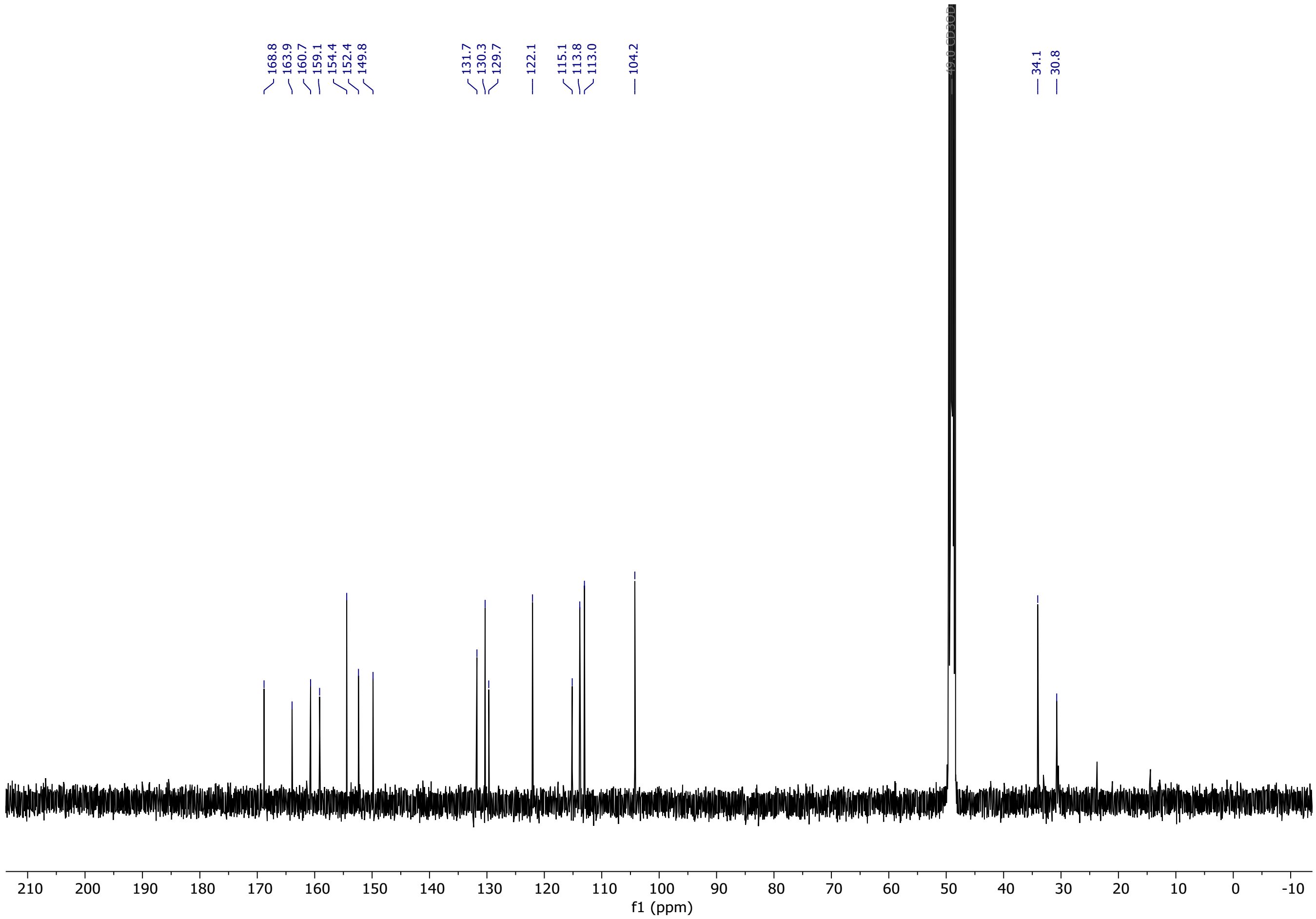

Figure S5. ^{13}C NMR spectrum of brazilein type B (**8**) in CD_3OD (126 MHz).

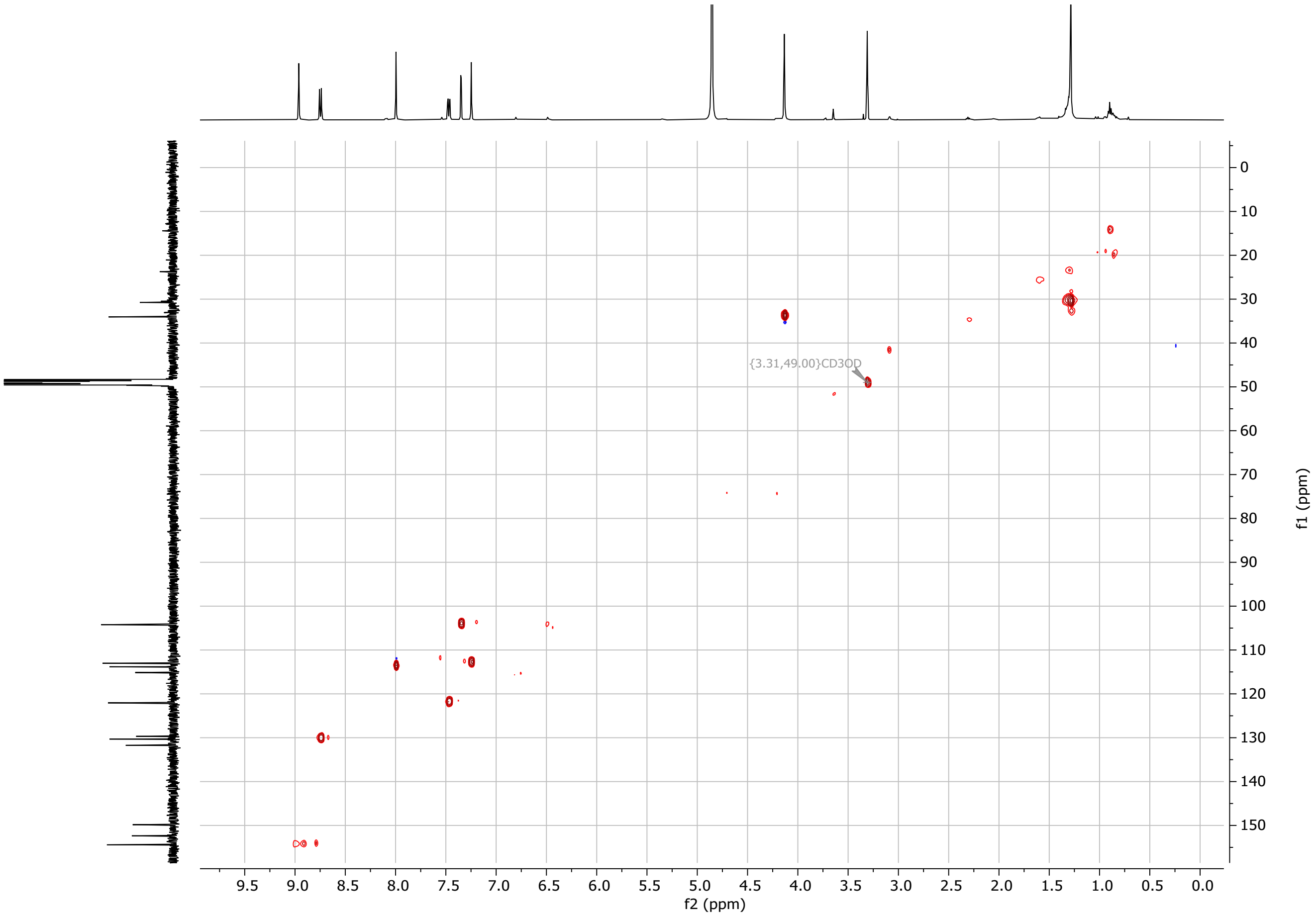

Figure S6. 2D HSQC NMR spectrum of brazilein type B (**8**) in CD_3OD.

.

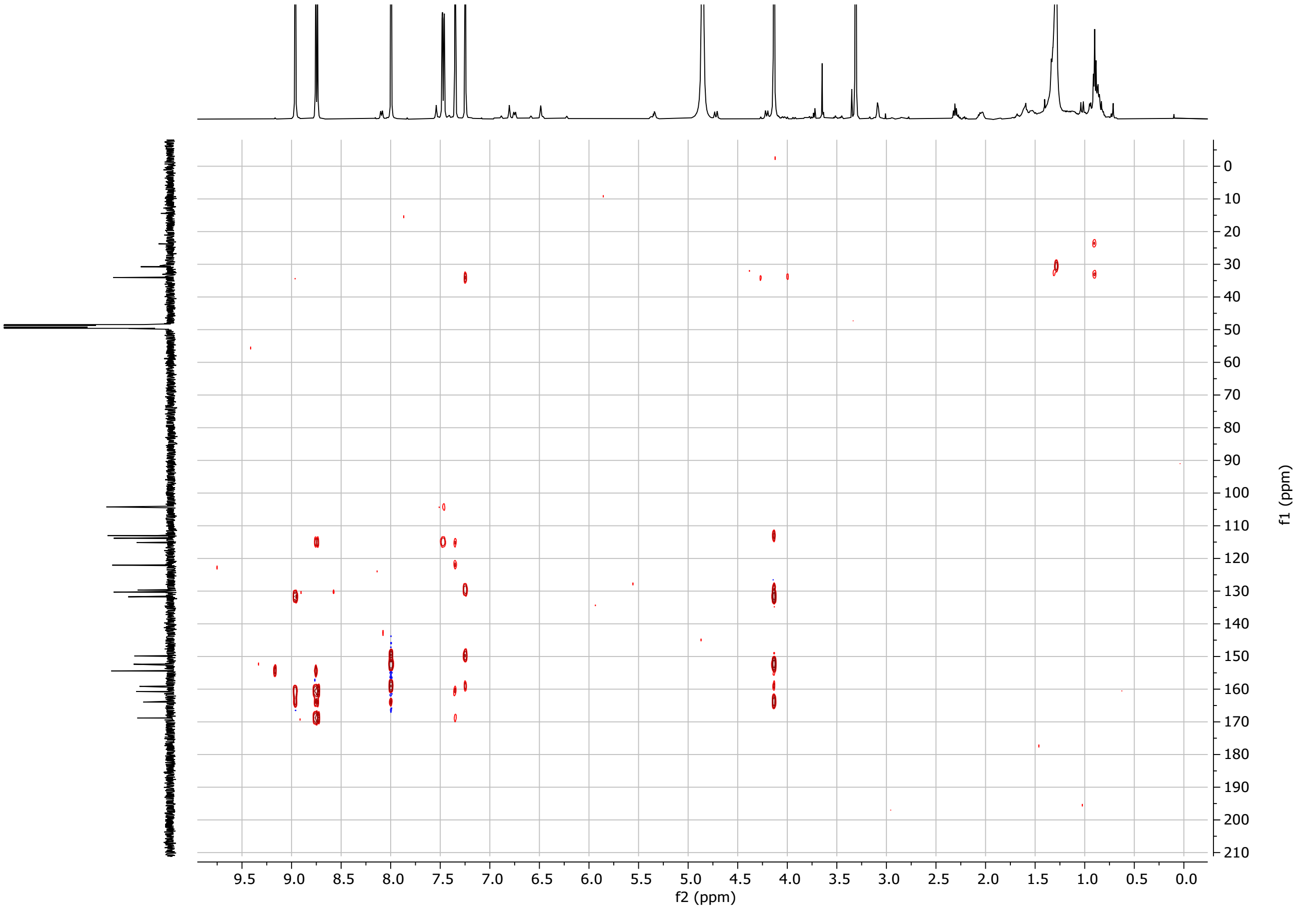

Figure S7. 2D HMBC NMR spectrum of brazilein type B (**8**) in CD_3OD.

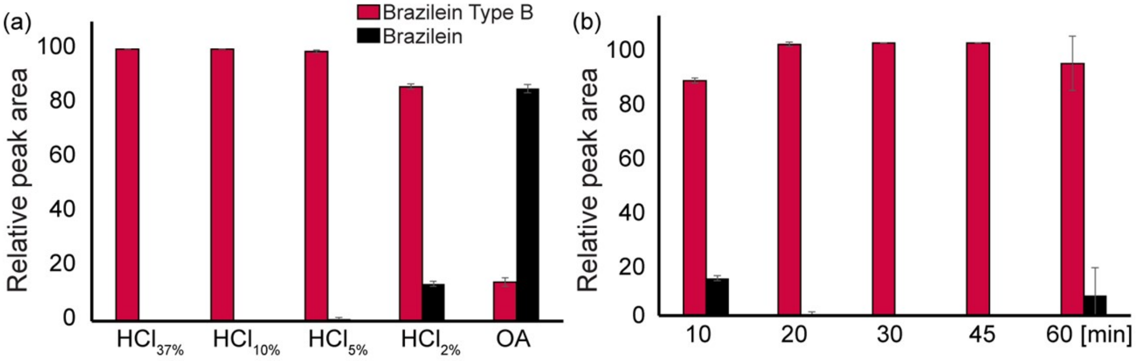

Figure S8. Relative integrated surface area monitored at 450 nm of brazilein (**3**) (black) and brazilein type B (**8**) (dark red) compounds extracted from wool samples (n=3) dyed with *Caesalpinia sappan* Brazilwood after variation of (a) acid strength HCl, 10 min at 100°C and OA 60 min at 80°C (b) time with 2% HCl at 100°C.

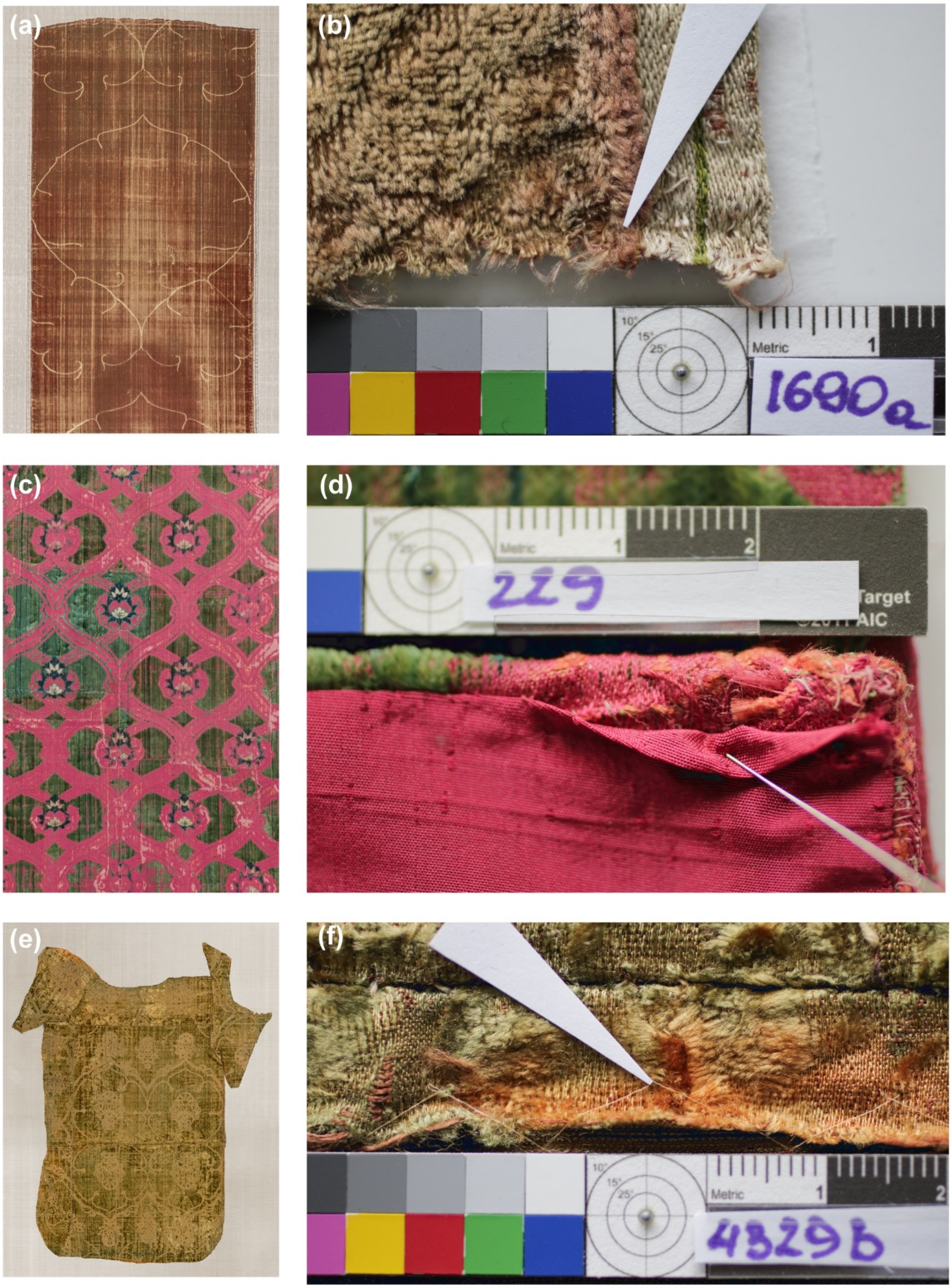

Figure S9. Sampling of Italian silk velvets dated between the 15-16th century from the Abegg-Stiftung's collection, of objects inv. no. 1690a resp. 229, and 4329b from top to bottom. General views of the objects (a, c, d) © Abegg-Stiftung, CH-3132 Riggisberg, 2014 (photo: Christoph von Viràg) and sampling details (b, d, f) © Abegg-Stiftung, CH-3132 Riggisberg, 2023 (photo: Textile conservation studio)

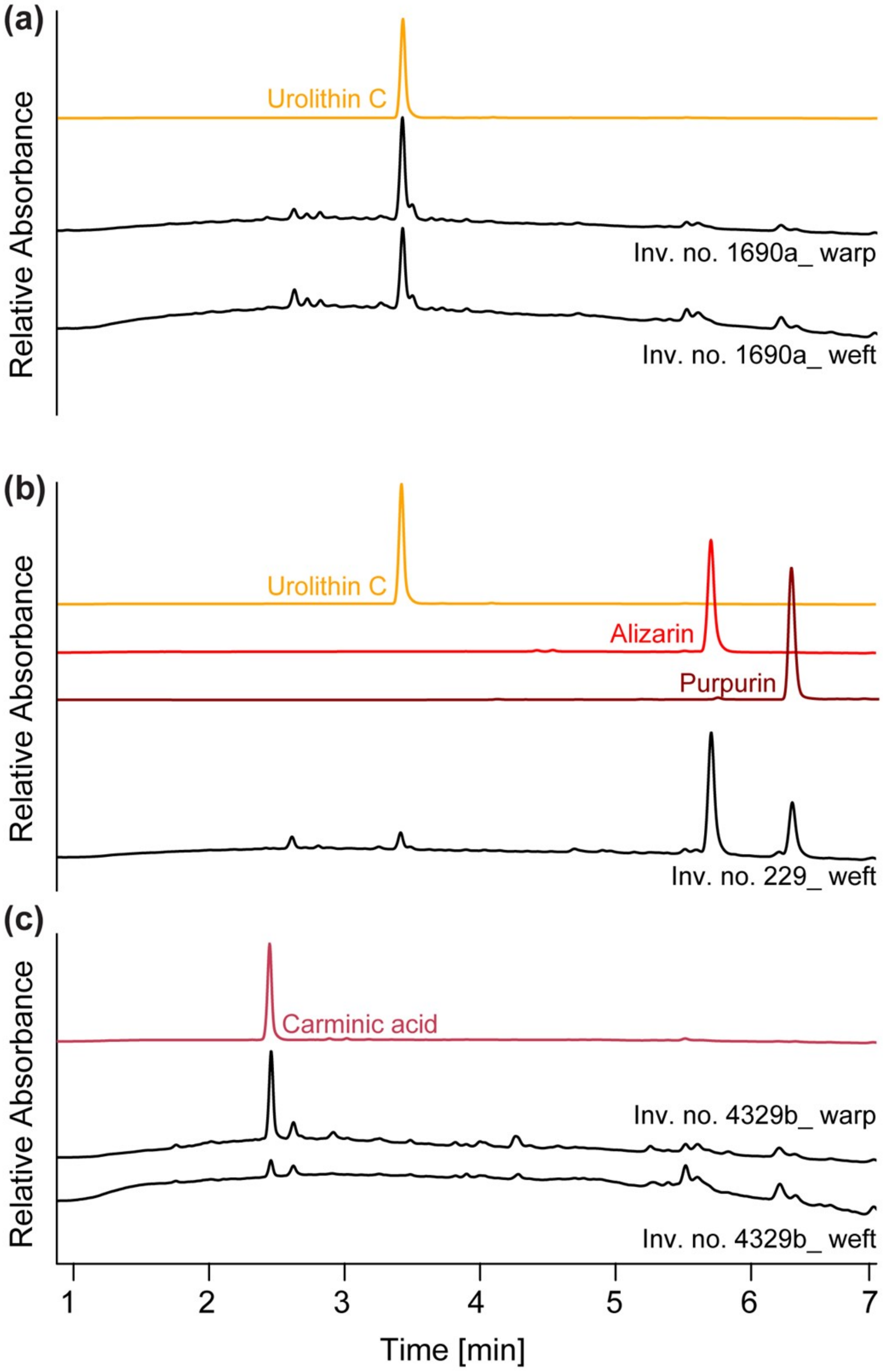

Figure S10. Chromatograms at 254 nm of extracts of sampled silk yarns in black from (a) object Inv no. 1690a, (b) Inv. no. 229 and (c) Inv. no. 4329b. Overlayed chromatograms are reference red standards.
